# Environmental determinants for participation among stroke survivors in Africa, a scoping review

**DOI:** 10.3389/fresc.2023.1136742

**Published:** 2023-05-23

**Authors:** Yohannes Awoke Assefa, Zelalem Dessalegn Demeke, Sara Wolde, Yisak Girma Guadie

**Affiliations:** ^1^ Occupational Therapy Department, School of Medicine, University of Gondar, Gondar, Ethiopia; ^2^ Occupational Therapy Student, Queen's University, Kingston, ON, Canada; ^3^ Physiotherapy Department, School of Medicine, University of Gondar, Gondar, Ethiopia

**Keywords:** stroke, participation, environmental factor, Africa, international classification of functioning (ICF)

## Abstract

**Purpose:**

In this review, we aimed to determine the environmental factors that are influencing the participation of stroke survivors in Africa.

**Methods:**

Four electronic databases were systematically searched from inception to August 2021, and identified articles were screened by the two authors of this review based on predetermined criteria. No date restrictions were imposed, and we included any type of paper, including gray literature. We followed the scoping review framework by Arksey and O'Malley, which was later revised by Levac et al. The whole finding is reported using the preferred reporting items for systematic reviews and meta-analyses extension for scoping reviews (PRISMA-ScR).

**Results:**

A total of 584 articles were generated by the systematic search, and one article was added manually. After eliminating the duplicates, the titles and abstracts of 498 articles were screened. From the screening, 51 articles were selected for full article review, of which 13 met the criteria to be included. In total, 13 articles were reviewed and analyzed based on the international classification of functioning, disability, and health (ICF) framework of the environmental determinants. Products and technology; natural environment and human-made changes to environment; and services, systems, and policies were found to be barriers for stroke survivors to participate in their community. Conversely, stroke survivors are getting good support from their immediate family and health professionals.

**Conclusion:**

This scoping review sought to identify the environmental barriers and the facilitators that are determining the participation of stroke survivors in Africa. The results of this study can serve as a valuable resource for policymakers, urban planners, health professionals, and other stakeholders involved in disability and rehabilitation. Nonetheless, additional research is necessary to validate the identified facilitators and barriers.

## Introduction

Stroke is one of the main causes of serious long-term disability worldwide (3). It is the second prevalent cause of death and a leading cause of disability in adults (4–6). Two-thirds of stroke cases occur in low- and middle-income countries (LMICs) (5). Also, over 87% of disability-adjusted life years (DALYs) from stroke were estimated to be in LMICs, which is about seven times the DALYs lost in high-income countries (7). The community-based studies conducted in Africa have shown that the age-standardized annual stroke incidence rate can be as high as 316 per 100,000 individuals, while the age-standardized prevalence rate can reach up to 981 per 100,000 individuals (8). The stroke incidence in low- and middle-income countries are continuously increasing (9), including in African countries (10).

Depending on the severity of the stroke and the area of the brain affected, stroke causes temporary or permanent impairments in motor, cognitive, speech, perceptual, and sensory skills (11). These impairments, when combined with environmental barriers, can significantly impede the ability of a stroke survivor to function and participate in life activities (12, 13).

Participation is defined as the “involvement in a living situation” (p. 14) in the international classification of functioning, disability, and health (ICF), and participation restrictions are described as problems an individual may experience in the involvement in life situations (p. 14) (14). Studies have shown that stroke survivors result considerable participation restrictions, such as inability to return to previous occupations, decreased social interactions, and inability to participate in religious and leisure activities (15, 16). Participation restriction has a significant negative impact on the health, quality of life, and wellbeing of stroke survivors (17). Both personal and environmental factors determine the level of participation and engagement of individuals who have experienced a stroke. Personal factors such as impairment, the severity of disabilities, and age can hinder social participation among stroke survivors. Furthermore, environmental factors such as the accessibility of the built environment, the cost of rehabilitation, and the level of social support can also have an impact on the involvement and participation of stroke survivors in various life situations. Therefore, it is important to consider both personal and environmental factors when designing interventions aimed at improving the social participation and quality of life of stroke survivors (18, 19).

Taking into consideration the unique lifestyle, varied culture, and socioeconomic status of people in Africa, we find it important to assess the environmental factors affecting the participation of people living with stroke in Africa. We aimed to explore the available literature to show the environmental factors that are affecting the participation of stroke survivors. Hence, we conducted this scoping review with the goal of comprehensively examining and synthesizing the environmental factors that positively or negatively affect the participation in life activities of stroke survivors in African countries.

## Materials and methods

The aim of this study was to analyze available literature so as to identify the environmental factors that are positively or negatively determining the participation of stroke survivors living in Africa. We used the framework suggested by Arksey and O'Malley (1), and later revised by Levac et al. (2), to complete this review. We reported our research based on preferred reporting items for systematic reviews and meta-analyses (PRISMA) extension for scoping reviews (20). Arksey and O'Malley (1) suggest five stages of conducting a scoping review: identifying the research question; identifying the relevant studies; study selection; charting the data; and collating, summarizing, and reporting the results (1).

Articles with various methods, including gray literature, were included in the review, and a time limit was not set in our search so we could include as many studies as we could. We presented our findings by group based on the environmental factors stated in the ICF model, namely, products and technology; natural environment and human-made changes to environment; support and relationships; attitudes; and services, systems, and policies.

### Identifying the research question

Our research question was “What are the environmental determinants for participation in stroke survivors who live in Africa?”

### Identifying relevant studies

We searched CINHAL, PubMed, Medline, and EMBASE. We also conducted a manual search on Google scholar. We searched the databases from inception to August 2021 so as to include all relevant available literature. We used the following keywords and subject heads: “(participation or engagement or involvement or participate) AND (challenges or barriers or difficulties or limitations or obstacles) AND (stroke or cva or cerebrovascular accident or hemiplegia or hemiparesis or poststroke or post-stroke or stroke survival) AND (Africa or sub saharan africa or african countries or Algeria or Angola or Benin or Botswana or Burkina Faso or Burundi or Cameroon or Cabo Verde or Central African Republic or Chad or Comoros or Congo or the Democratic Republic of Congo or Cote dIvoire or Djibouti or Equatorial Guinea or Egypt or Eritrea or Ethiopia or Gabon or Gambia or Ghana or Guinea or Guinea-Bissau or Kenya or Lesotho or Liberia or Libya or Madagascar or Malawi or Mali or Mauritania or Mauritius or Morocco or Mozambique or Namibia or Niger or Nigeria or Rwanda or Senegal or Seychelles or Sierra Leone or Somalia or South Africa or Sudan or Swaziland or Tanzania or Togo or Tunisia or Uganda or Zambia or Zimbabwe).”

### Study selection

We exported the endnote citation and performed the screening process using the Covidence software. A total of 585 articles were retrieved from the databases after eliminating the duplicates. The titles and abstracts of 498 articles and, consequently, the full articles of 51 studies were reviewed by the two authors (YAA and ZDD), of which 13 articles met the criteria and were included in the scoping review. Conflicts were discussed between the two authors until a consensus was reached. The full articles of the 13 included studies were imported into NVivo 12 plus software for easy access to reading and synthesizing. We used the ICF framework (14) to thematize and present the review. The ICF framework, under the environmental factors, comprises products and technology; natural environment and human-made changes to environment; support and relationships; attitudes; and services, systems, and policies (14).

**Figure 1:**
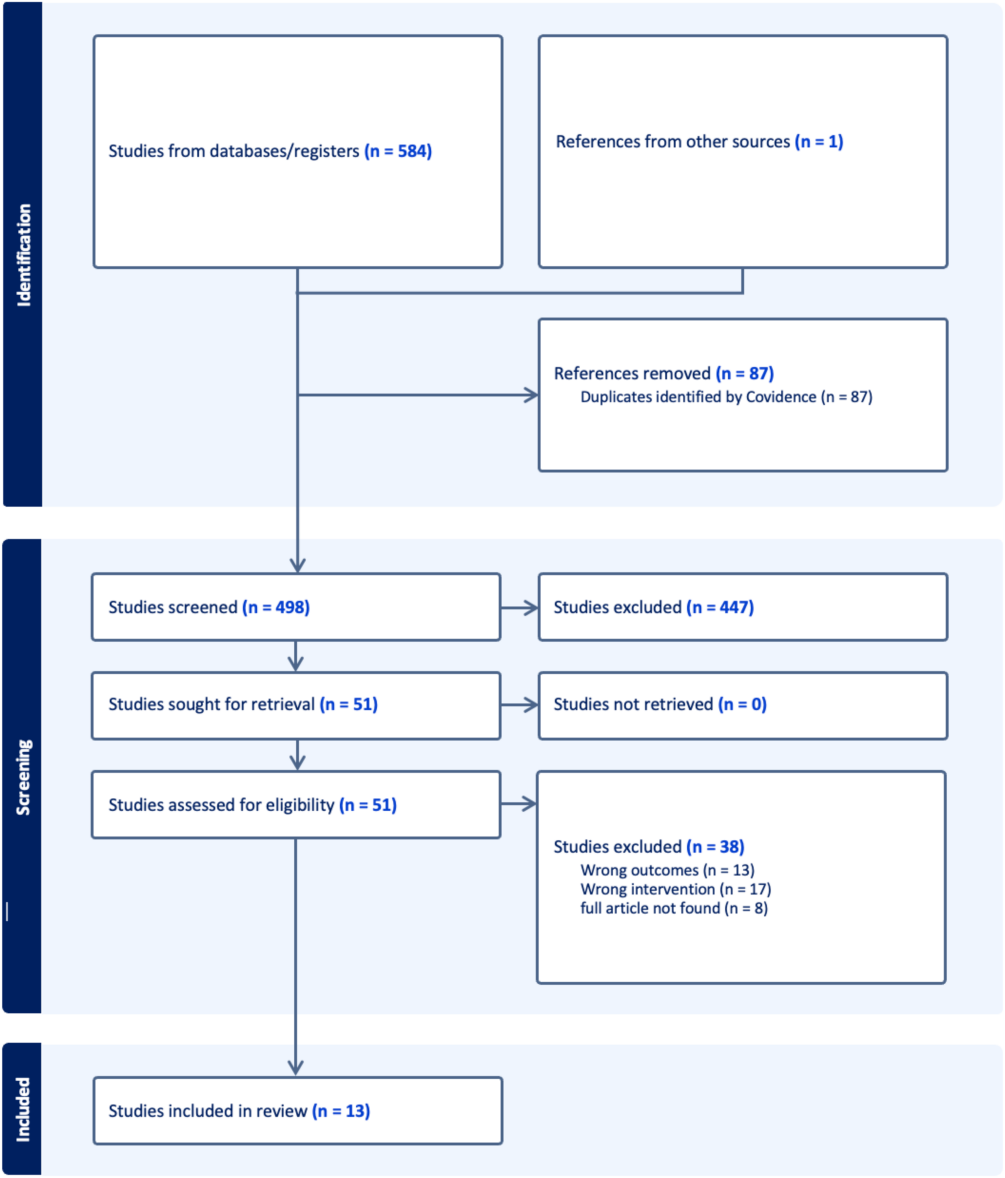
Flowchart of article selection.

Figure 1 shows the PRISMA diagram of the process used in the paper selection. Studies based on the following predetermined criteria were included:

• Articles only in English.
• All style literature, including gray literature, was included to maximize and broaden the scope of literature.
• Studies conducted in Africa.
• Studies that assessed the environmental determinants of stroke survivors.
• All age group populations who survived stroke, not transient ischemic attack (TIA).

## Results

A total of 13 studies met the eligibility criteria and were included in the scoping review. Table 1 summarizes the characteristics of the included studies. The majority of the studies were conducted in South Africa.

**Table 1:** Study characteristics of all included studies.

	Author(s)	Year of publication	Study location	Study populations	Aims of the study	Methodology
1.	Cawood and Visagie, 2015	2015	South Africa	Adult stroke survivors	To determine environmental barriers and facilitators to participation experienced by a group of stroke survivors in the Western Cape province of South Africa	Mixed method
2.	Amosun et al., 2013	2013	Ghana	Adult stroke survivors	To assess the perceived and experienced restrictions in participation and autonomy among adult stroke survivors in Ghana	Mixed method
3.	Mudzi et al., 2013	2013	South Africa	Adult stroke survivors admitted to hospital for ischemic stroke	The aim of this study was to establish the level of community participation of patients at 12 months post-stroke and the associated factors impacting on that participation	Mixed method
4.	Vincent-Onabajo et al., 2016	2016	Nigeria	Adult stroke survivors	To investigate the impact of social support on participation of stroke survivors in Nigeria	Quantitative
5.	Maleka et al., 2012	2012	South Africa	Adult stroke survivors	The aim of this study was to establish the experience of people living with stroke in low socioeconomic urban and rural areas of South Africa	Qualitative study
6.	Rhoda et al., 2015	2015	South Africa	Adult stroke survivors	The aim of this paper is to present the provision of in-patient stroke rehabilitation. In addition, the challenges experienced by the individuals with participation post discharge are also presented	Mixed method
7.	Elloker et al., 2019	2019	South Africa	Adult stroke survivors	The aim of this study is to investigate the factors influencing community participation among community-dwelling stroke survivors in the Western Cape, South Africa	Quantitative method
8.	Arowoiya, 2014	2014	South Africa	Adult stroke patients	The aim of this study was to determine and explore the participation restrictions experienced by stroke patients	Mixed methods
9.	Elloker, 2016	2015	South Africa	Adult stroke patients	The aim of this study is to determine social support and participation restrictions in patients with stroke living in selected areas in the Western Cape	Quantitative method
10.	Ekechukwu et al., 2017	2017	Nigeria	Adult stroke patients	This study aims to investigate the clinical and psychosocial predictors of community reintegration among stroke survivors 3 months post in-hospital discharge	Qualitative method (exploratory study)
11.	Urimubenshi, 2015	2015	Rwanda	Stroke patients	To explore the activity limitations and participation restrictions experienced by people with stroke in Musanze district in Rwanda	Qualitative method (phenomenological)
12.	Soeker and Olaoye, 2017	2017	Nigeria	Stroke survivors	The study was aimed at exploring and describing the experiences of rehabilitated stroke survivors and perceptions of stakeholders about stroke survivors returning to work in South-West Nigeria	Qualitative method
13.	Rhoda, 2012	2012	South Africa	Stroke survivors	The aim of the study was to explore the activity limitations and participation restrictions experienced by patients with a stroke	Qualitative method

### Product and technology

Product and technology is defined by WHO as “any product, instrument, equipment or technical system used by a disabled person, especially produced or generally available, preventing, compensating, monitoring, relieving or neutralizing” disability (14).

Five studies mentioned how products and technologies facilitate or hinder participation of people living with stroke. In the study by Cawood and Visagie (21), 77% of participants identified products and technology as a barrier. Difficulty in frequently using transport appeared as a barrier (21–24). In their study, Mudzi et al. (23) revealed that 100% of stroke survivors mentioned transport services as mild to moderate barriers to participation. Cawood and Visagie (21) also mentioned in their study that inaccessible transport creates a barrier for 80% of the community participation of stroke survivors (21).

In addition, it was stated that people with stroke need to pay extra to use public transport if they could find a driver who is willing to take them (22, 23). The independence of individuals with stroke in accessing private transportation is limited due to the lack of accessibility of public transport. Consequently, these individuals are often compelled to rely on others to assist them in getting in and out of the car, which can sometimes compromise their dignity (22). Lack of assets such as money also appeared as a barrier to participation in social activities (21). Cawood and Visagie (21) stated that stroke survivors were not able to afford for phone service. Not only this, but they were also unable to pay for assistive devices (21). In the same vein, access to and utilization of assistive devices were also found to be low in the studies (21, 22). Cawood and Visagie (21) stated that even though most people with stroke can access mobility devices, it is difficult for them to get other assistive products such as bath transfer, grab bars and ankle foot orthosis. For example, it was mentioned that people with stroke struggle to use a toilet that is not modified to accommodate their need (22).

### Natural environment and human-made changes to environment

Regarding natural environment and human-made changes to environment, nine articles met the inclusion criteria (15, 21, 22, 24–29). Almost all studies concluded that the natural and human-made environment is inaccessible and creates barriers to participation for people with stroke living in Africa. Amosun et al. (25) stated in their article that environmental barriers led to self-imposed restrictions as stroke survivors would prefer to stay at home than go out and experience the environmental difficulty.

Human-made changes to the environment prevent stroke survivors from participation, including participating in therapy. Cawood and Visagie (21) found in their study that 65% of public buildings were inaccessible. Inaccessibility of the environment hindered people living with stroke from participating in rehabilitation therapy (27, 28). This further complicated their condition and deteriorated their recovery and ability to participate. Soeker and Olaoye (27) concluded that the distance from home to clinic was a major factor not to adhere to therapy. The inaccessibility forced some people to change their home address to live near to hospitals where they get therapy (15). This led them to lose their previous social contact from their old neighborhood (15). Not only the public buildings and the neighborhood, the home environment of stroke survivors was also inaccessible. Maleka et al (29) revealed that the homes were small and cluttered.

Natural environments also imposed restrictions on participation. Walking or pushing a wheelchair on sandy and uneven pavements creates a huge inaccessibility and results in hindered social participation (21). Sandy and uneven pavements in the neighborhood make mobility, with or without a wheelchair, very difficult (15, 21). Arowoiya (22) also asserted that among their study participants, about 21% face severe difficulty in dealing with the physical environmental barriers in their society. Also, Rhoda et al. (28) reported that stones on the way, stairs, and uneven grounds create a barrier to using wheelchair and hinder social participation of stroke survivors.

### Support and relationships

A total of 11 articles out of 13 discussed support and relationships in stroke survivors. We found a contradicting result. Six articles (21, 23, 27, 30–32) discussed people living with stroke are getting positive social support that is facilitating participation. Conversely, five articles (15, 22, 24, 25, 28) stated that social support and relationships were low and eventually negatively affected stroke survivors.

Mudzi et al. (23) assessed the support and relationship in terms of the immediate family, personal care providers, friends, acquaintances, peers, colleagues, neighbors, and community members. They found that immediate family and personal care providers were supportive and facilitators of participation (23). However, stroke patients perceived that the lack of support from their friends is a barrier to social participation (23). Cawood and Visagie (21) found that the majority (88%) of the immediate families of stroke survivors were supportive. Stroke survivors need and get social support and assistance for activities of daily living (ADL) and instrumental activities of daily living (IADL) from family members (32). By the same token, they also get positive support in the workplace to resume their previous work (27). Ekechukwu et al. (30), while assessing the clinical and psychosocial predictors of community reintegration of stroke survivors, revealed that stroke survivors who received good social support were better at reintegrating into the community. Vincent-Onabajo et al. (31) also asserted that a high level of social support is associated with better social participation and economic self-sufficiency.

However, we also understood from the articles mentioned that as time passes, the support and relationship diminishes (28). In the study done in Rwanda, to assess the activity limitations and participation restrictions, Urimubenshi (15) revealed that the social interaction of stroke survivors decreased from time to time. One reason stated was that people with stroke frequently change their residence near to hospital as it would be easier for them to get continuous healthcare services (15), which leads to the lack of support from their previous social capital. Others also could not maintain their relationship with friends due to financial restrain (22). Elloker (24) assessed the social support and participation restrictions in patients living with stroke in South Africa. They revealed that nearly 90% of stroke survivors have low social support (24). Lack of adequate social support diminishes participation (25, 28). However, stroke survivors value the support they receive (24).

### Attitudes

Four articles discussed how the attitude toward stroke survivors positively or negatively determines participation (21–23, 25).

Cawood and Visagie (21) found in their study that the majority of immediate families have positive attitudes toward stroke survivors. In addition, the attitude of health professionals was a facilitator for participation (21). However, the societal attitude was found to be negative and created a barrier to participation (21, 22). Mudzi et al. (23) also revealed in their study that the majority of the attitudes of friends were a barrier for stroke survivors to participate in their community.

We also understood from the articles that people see stroke survivors as pitiful and support them from a sense of duty (21, 22). The negative attitude does not always come from another person, but stroke survivors also have a perceived negative attitude that hinders their participation (25). Amosun et al. (25) concluded that stroke survivors experienced both self and enacted stigma.

### Services, systems, and policies

We could not find enough information about services, systems, and policies and how they are affecting the participation of stroke survivors. Three articles (21, 23, 27) discussed services, systems, and policies regarding participation. These factors appeared to be barriers to participation for stroke survivors. For example, Cawood and Visagie (21) revealed that nearly half of their study participants indicated that they did not receive assistance from associations or organizations. Cawood and Visagie (21) and Mudzi et al. (23) presented that both the housing policies (23) and the bureaucracy to get government-subsidized houses create barriers. In addition, the paperwork to process disability benefits took too long, which led to financial strain (21). In another study, Soeker and Olaoye (27) indicated that stroke survivors struggle with financial constraints that lead them to opt out from therapy. This indicates that there was minimum or no support to help them continue their therapy.

## Discussion

Evidence has shown that personal factors such as level of function, motor activity, cognitive ability, and executive function determine the level of participation (33–35). However, the level of participation can also be determined by environmental factors. The environment of African countries is different from that of western countries. In this review, we particularly examined the environmental determinants of participation among stroke survivors living in African countries.

Regarding product and technology, access to transport, service charge for transportation, and access to assistive products appeared to be a barrier to participation for stroke survivors (21–24, 32). Stroke survivors struggle to get accessible public transport, and even if they get access, they have to pay extra for the service, and they will have to get assistance as well (22, 23). It was also evident from the studies that there is limited access to assistive devices that limit mobility in the community, which eventually limits participation. The low provision and utilization of products and technologies, as it was evident from the literature, greatly hinders the participation of stroke survivors in life activities. Rhoda (32) mentioned how stroke survivors can benefit from and are dependent on their walking devices. Being unable to get assistive devices creates frustration in going out and participating in the community. Hence, people usually prefer to stay at home and avoid social participation. This indicates that increasing the accessible transport system and access to assistive technologies could help to facilitate the participation of stroke survivors.

The natural and human-made environment was also found inaccessible and hinders participation. It is obvious that a fully accessible environment facilitates mobility and participation (26). Inaccessible environments can impede the participation of stroke survivors in various areas of life, such as obtaining healthcare services (25, 26). As a result, they may choose to restrict their own activities, leading to a decline in their overall wellbeing (31). Although mobility in a fully accessible environment facilitates participation (26), the result from the reviewed articles showed that the road in the neighborhood of stroke survivors was full of obstacles and their home address was far from hospitals (15, 21, 28). In addition, public buildings were also found to be inaccessible, and people with mobility issues could not access (21). Based on the findings of the reviewed articles, it is recommended that efforts should be made to improve the accessibility of both the natural and human-made environment for stroke survivors. This can be achieved through a combined effort of policymakers, urban planners, architects, transportation authorities, and healthcare providers.

A lot of evidence, even though contradicting to each other, was found regarding support and relationships. A total of 11 articles out of 13 discussed how support and relationships are positively or negatively affecting participation among stroke patients in Africa. The articles (21, 23, 27, 30–32) asserted that stroke survivors receive good social support from immediate family, clinicians, and coworkers but not adequate support from friends (23). However, it was also evident from the articles that the support diminishes as time passes (28). Support from friends is just as important as support from other concerned parties in facilitating the recovery and wellbeing of stroke survivors. Many people prefer engaging in leisure activities with friends rather than family members, highlighting the significance of fostering supportive friendships. As leisure activities are an essential part of human life, it is important to make efforts to enhance the support and relationships with friends. In general, it is better if family members and friends get professional advice on how to provide support for stroke survivors.

Similarly, the attitude of immediate family and health professionals was found to be positive and facilitating (21). However, the negative societal attitudes are barriers to participation (21, 22). Stroke survivors also had a negative attitude about themselves that caused self-induced participation restriction (25). This can also be addressed by educating stroke survivors and the community about the condition (i.e., stroke) and the disability in general to promote participation.

There was a scarcity of evidence showing the effect of services, systems, and policies on participation. The existing evidence, however, shows the services, systems, and policies are barriers to participation (21, 23, 27). There is limited assistance from government or non-government organizations for stroke patients (21).

The limitations of this review were the following: we only included articles published in the English language. In addition, a methodological appraisal was also beyond the scope of this study. Hence, its absence can be considered as a limitation of the study.

## Conclusion

In conclusion, this scoping review sought to identify the environmental barriers and the facilitators that are determining the participation of stroke survivors in Africa. Products and technology; natural environment and human-made changes to environment; and services, systems, and policies are found to be barriers for stroke survivors to participate in their community. Conversely, stroke survivors are getting good support from their immediate family and health professionals. The results of this study can serve as a valuable resource for policymakers, urban planners, health professionals, and other stakeholders involved in disability and rehabilitation. Nonetheless, additional research is necessary to validate the identified facilitators and barriers.
